# BRAT1 Mutation Retrospective Diagnosis: A Case Report

**DOI:** 10.7759/cureus.35655

**Published:** 2023-03-01

**Authors:** Fabiana Vercellino, Massimo Valerio, Maria Pia Dusio, Alice Spano, Sandra D'Alfonso

**Affiliations:** 1 Child Neuropsychiatry Unit, SS Antonio e Biagio e Cesare Arrigo Hospital, Alessandria, ITA; 2 Pediatric Intensive Care Unit, SS Antonio e Biagio e Cesare Arrigo Hospital, Alessandria, ITA; 3 Clinical Genetics, Clinical Biochemistry Laboratory, Maggiore della Carità Hospital, Novara, ITA; 4 Department of Health Sciences, University of Eastern Piedmont, Novara, ITA

**Keywords:** brat1 gene sequencing, brat1 mutation, neonatal hypertonia, epileptic encephalopathy, lethal neonatal rigidity

## Abstract

Biallelic mutations in the BRAT1 gene have been reported in cases with Lethal neonatal rigidity and multifocal seizure syndrome (RMFSL), since 2012. Clinical features include progressive encephalopathy, dysmorphic features, microcephaly, hypertonia, developmental delay, refractory epilepsy, episodic apnea, and bradycardia. More recently, biallelic BRAT1 mutations have been associated with a milder phenotype in patients with migrating focal seizures in the absence of rigidity or with nonprogressive congenital ataxia with or without epilepsy (NEDCAS). It has been proposed that the loss of function caused by BRAT1 mutations may decrease cell proliferation and migration and cause neuronal atrophy through impairment of mitochondrial homeostasis. We here report a female infant with a phenotype, electroencephalogram (EEG), and brain magnetic resonance imaging (MRI) consistent with RMFSL, whose diagnosis was indirectly formulated three years after death upon the identification in both parents of a known pathogenetic variant in the BRAT1 gene. Our report emphasizes the remarkable potential of novel genetic technologies for the diagnosis of past unsolved clinical cases.

## Introduction

Biallelic mutations in the BRAT1 (BRCA1-ASSOCIATED ATM ACTIVATOR 1) gene on chromosome 7p22 have been reported in patients with a rare autosomal recessive syndrome characterized by lethal neonatal rigidity and multifocal seizure (RMFSL, MIM #614498) since 2012 [1-4]. Subsequent descriptions of affected patients have helped to delineate this rare progressive encephalopathy characterized by dysmorphic features (microcephaly, hyper or hypotelorism, microphthalmia, smooth, and long philtrum), hypertonia, refractory seizures, and dysautonomia (hypothermia, apnea, bradycardia, sudden infant death syndrome-SIDS) requiring intubation and eventually death in the first years of life [5-7]. Mildly affected individuals with BRAT1 mutations were reported [8-10] with a phenotype known as a neurodevelopmental disorder with cerebellar atrophy and with or without seizures (NEDCAS, MIM# 618056). The neuropathological evaluation reported microcephaly, severe neuronal loss, and background gliosis in the dorsal region of the putamen. The loss of function mutations associated with RMFSL syndrome has been proposed to impair the DNA damage response pathway and mitochondrial homeostasis [5]. We here report a female infant with non-consanguineous Romanian parents with a phenotype, electroencephalogram (EEG) test, and brain magnetic resonance imaging (MRI) consistent with RMFSL, whose diagnosis was achieved three years after death by performing the genetic analysis on the parents’ blood samples.

## Case presentation

**Figure 1:**
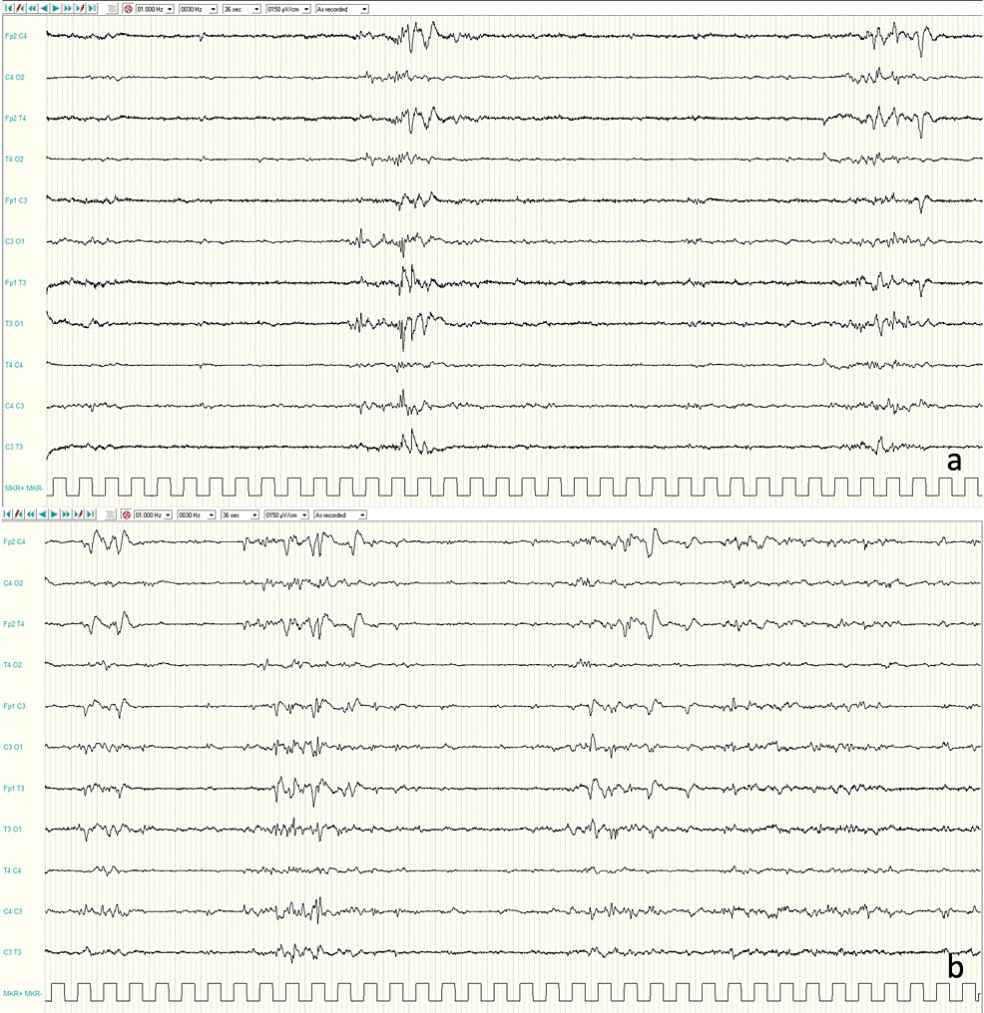
Interictal EEGs Awake and sleep EEGs showed a burst-suppression pattern and medium-high voltage multifocal spikes and sharp waves especially over temporal and central regions (a-b).

The girl was the first child of non-consanguineous Romanian parents. She was born at term by caesarian section following a pregnancy complicated by intrauterine growth restriction and polyhydramnios. The amniocentesis result was normal. The newborn’s APGAR scores were 4, 6, and 7, assessed at 1, 5, and 10 minutes, respectively. The birth weight was 2.400 g (10th percentile), and the head circumference was 29.4 cm (below the 3rd percentile). She was admitted to our Neonatal Intensive Care Unit, and shortly after birth, she showed dysmorphic features, hypertonia, and frequent myoclonic and clonic seizures involving her face and limbs. Serum glucose, ions, lactate, ammonia, and liver enzymes were normal. The septic workup was negative. Urinary organic acids and serum quantitative amino acids showed no abnormalities. After informed consent was obtained from her parents, genetic tests (Karyotype and array comparative genomic hybridization analysis) on the peripheral blood of the patient were performed, demonstrating no pathogenetic variants. EEGs showed a burst-suppression pattern with medium-high voltage multifocal spikes and sharp waves, especially over the temporal and central regions, background slowing, and no posterior rhythm (Figure 1a-1b).

**Figure 2:**
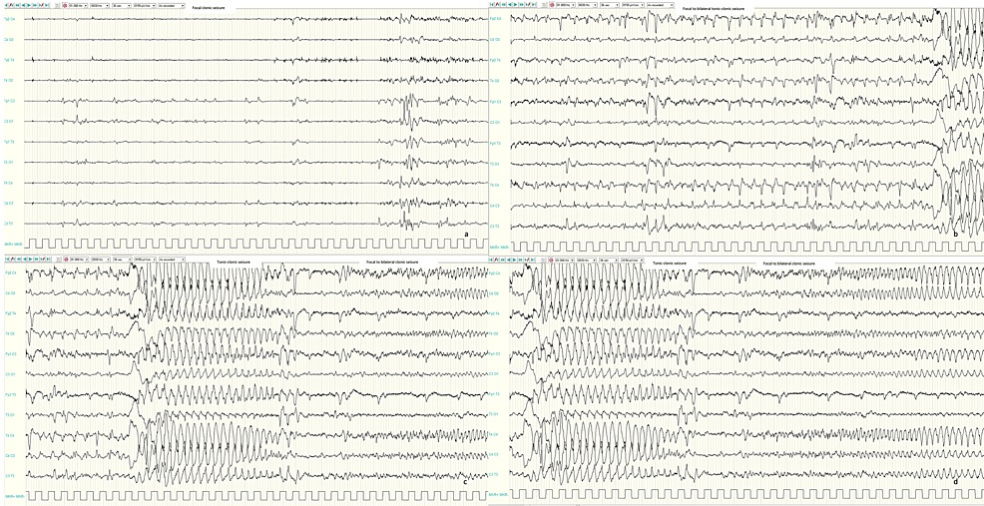
Ictal EEGs Ictal EEGs showed focal repetitive sharp waves on left central region corresponding to a focal clonic seizure (a); focal to bilateral repetitive sharp waves on centro-temporal regions (especially on the right) followed by generalized high amplitude spike and slow-wave discharge corresponding to a focal to bilateral tonic-clonic seizure (b-d).

From day 3 of life, focal myoclonic and clonic seizures with or without apnea were observed, while from week 2 of life, focal to bilateral tonic, and tonic-clonic seizures appeared many times a day. Ictal EEGs showed focal repetitive sharp waves (Figure 2a) or focal to bilateral repetitive sharp waves followed by generalized high amplitude spikes and sharp wave discharge (Figure 2b-2d).

**Figure 3:**
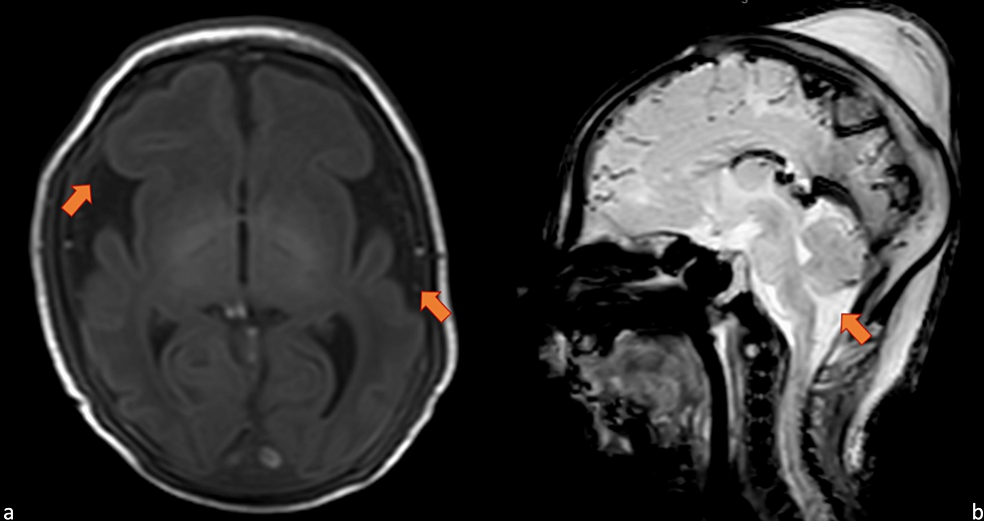
Brain MRI on day 7 of life Brain magnetic resonance imaging on day 7 of life. Frontotemporal oligogyric microcephaly and mild prominence of the pericerebral CFS space on axial T1-weighted imaging (a), large pericerebellar cisterns and cerebellar hypoplasia on sagittal T2-weighted imaging (b).

The echocardiogram and eye examination were normal. MRI examination demonstrated microcephaly with a simplified fronto-temporal gyral pattern or oligogyric microcephaly, mild prominence of the pericerebral cerebrospinal fluid (CSF) space, and large pericerebellar cisterns (Figure 3a-3b).

**Figure 4:**
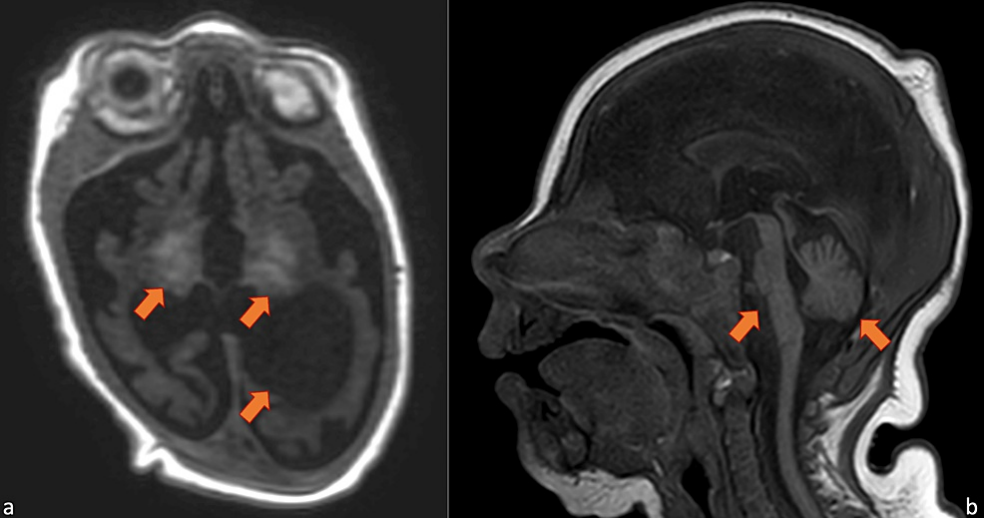
Brain MRI at follow up Brain magnetic resonance imaging on month 6 of life. Hydrocephalus, further cortical cerebral atrophy and focal hyperintensity in the basal ganglia on axial T1-weighted imaging (a); marked cerebellar and midbrain atrophy on sagittal T1-weighted (b).

Brain-malformation panel gene sequencing was tested to study 40 genes associated with brain malformations, and no pathogenic variants were observed. In the following weeks, the patient continued to suffer from intractable seizures and was treated with phenobarbital, pyridoxine, levetiracetam, phenytoin, topiramate, clonazepam, and midazolam with no effect. A pharmacological coma was started with thiopental obtaining a suppression-burst type trace in the absence of clinical or electrical crises. Five days after the discontinuation of thiopental, seizures restarted. The girl was unable to feed by mouth and had frequent spontaneous apnea and bradycardia episodes requiring intubation and mechanical ventilation. She was transferred to another hospital and underwent a tracheotomy and percutaneous endoscopic gastrotomy (PEG). Then she was re-transferred to our pediatric intensive care unit and continued to suffer from daily seizures. When she was six months old, another brain MRI was performed, showing an enlargement of the lateral ventricles (hydrocephalus), further cortical cerebral and cerebellar atrophy, and focal hyperintensity in the basal ganglia (Figure 4a-4b).

EEGs showed low amplitude, discontinued theta activity, and rare focal sharp waves. She was discharged at the age of six months, and she returned home. At her last EEG, when she was 11 months old, the trace showed disorganized, low-amplitude theta activity. She made no developmental progress and continued to suffer from epileptic seizures and bouts of apnea-bradycardia, culminating in cardiopulmonary arrest and death when she was 19 months. Suspecting a mutation in the BRAT1 gene, three years later the parents were re-contacted and offered to undergo a genetic analysis to attempt a retrospective diagnosis on their girl. Since no DNA sample was available for the affected girl, only recessive conditions could be informative for a retrospective indirect diagnosis with the identification of pathogenetic mutations in the parents. The analysis was conducted using whole exome sequencing (WES) with the Illumina NextSeq platform with target sequencing methodology on genomic DNA extracted from the whole blood of the parents, collected with their informed consent for genetic diagnostic and research analyses. The variant in BRAT1 was confirmed by the Sanger sequencing method. Sequencing results revealed the presence of a heterozygous variant, c.638dupA (p.Val214Glyfs*189), in exon 5 of the BRAT1 gene in both parents, classified as a "pathogenetic" variant according to the standard international guidelines [11]. Since mutations in BRAT1 are responsible for an autosomal recessive syndrome (Lethal neonatal rigidity and multifocal seizure syndrome - RMFSL), a retrospective diagnosis of epileptic encephalopathy due to the BRAT1 mutation was formulated, and the parents underwent genetic counseling.

## Discussion

In 2012, three Amish separate sibships carrying a homozygous mutation in the BRAT1 gene were first described by Puffenberger et al. [1]. Subsequently, a further 20 cases have been published (table in Van Ommeren et al. 2018) [5]. Another neonate with RMFSL was described in 2021 [7]. Clinical features included progressive encephalopathy, dysmorphic features (depressed frontal bones), microcephaly, severe axial and appendicular rigidity, developmental delay (ranging from severe to mild), refractory seizures, and dysautonomia (hypothermia, apnea, bradycardia, SIDS). Interictal EEGs showed background slowing, multifocal medium-high voltage spikes, and sharp waves, especially over the temporal and central regions, but no posterior rhythm. A severe form of epilepsy could be present with migrating focal seizures. Brain MRI showed cerebral/cerebellar atrophy or hypoplasia, impaired myelination, and thinning of the corpus callosum. Death usually occurs within the first years of life, although a few cases with a milder phenotype have been described [10]. In recent years, mutations in BRAT1 were discovered in patients with migrating focal seizures in the absence of rigidity or with nonprogressive cerebellar signs even in the absence of a neurodevelopmental disorder [12,13]. A wide range of homozygous or compound heterozygous BRAT1 mutations has been reported in affected children. The frameshift mutation in the BRAT1 gene detected in our family has already been reported in the literature as a pathogenic variant associated with RMFSL (MIM #614498), and in homozygous status, it has been associated with a particularly severe condition in comparison with other biallelic combinations of BRAT1 variants [1,10,14,15]. Even if we were not in the condition to confirm the presence of the BRAT1 biallelic mutation in the patient, the identification of a rare pathogenic mutation in both parents and the high consistency of the patient phenotype with literature on patients with BRAT1 mutations, strongly support a retrospective diagnosis of RMFSL for our patient. This variant was predicted to change the amino acid sequence after valine 214 and to introduce a premature stop codon 188 amino acid positions downstream, and it is very rare in the general population. Even if the parents did not report any consanguinity, they both came from the same small Romanian village, suggesting that they may have inherited the rare mutation from a common ancestor. The BRAT1 gene is located on chromosome 7p22.3 and encodes an 821 amino acid peptide, the BRCA1-associated ATM activation-1 protein (BRAT1) [16]. The authors suggested that BRAT1 has a role in stabilizing activated ATM protein following the DNA damage response. The role of BRAT1 in the DNA damage response is also suggested by its interaction with the BRCT (BRCA1 C Terminus) domain of the tumor suppressor gene BRCA1 and its binding to ATM1 and DNA-PKs [16-18]. Some authors speculated that the destabilization of the encoded protein may also underlie catastrophic epilepsy and corticobasal neuronal degeneration [19]. The loss of BRAT1 expression or function due to biallelic BRAT1 mutations may decrease cell proliferation and migration and cause neuronal atrophy through impairment of mitochondrial homeostasis [1]. The epilepsy of infancy with migrating focal seizures (EIMFS) is one of the most severe developmental and epileptic encephalopathies. This syndrome is identified by focal seizures with observed clinical and/or electrical migration between hemispheres and seizures onset before the age of six months. A genetic cause can be identified in a large number of patients with EIMFS [20]. In our experience, BRAT1 mutations could be suspected when EIMFS is associated with severe neonatal hypertonia.

## Conclusions

To date, no therapeutic modalities beyond supportive care have been developed for lethal neonatal RMFSL. Therefore, making a correct and prompt diagnosis of RMFSL in the patients and their families is extremely important to offer prompt genetic counseling to the family to evaluate prenatal diagnostic options and carrier testing for the prevention of the disease. We suggest the possibility of confirming the presence of BRAT1 mutations even in cases of past missed diagnoses throughout the genetic analysis performed on the parents’ blood samples.

This case emphasizes the remarkable potential of novel genetic technologies for the diagnosis of past unsolved clinical cases. Looking to the future, an early precise genetic diagnosis will allow the timely direction of patients to precision therapies.
